# A validated amplicon panel for parentage reconstruction and selection monitoring in whitefish (*Coregonus* spp.)

**DOI:** 10.1007/s12686-026-01449-2

**Published:** 2026-10-16

**Authors:** Caleb Beck, Marion Leleu, Marina Brasó‐Vives, Stéphane Daly, Sandesh Shrestha, Grégory Brazzola, Claus Wedekind

**Affiliations:** 1 Department of Ecology and Evolution, University of Lausanne, 1015 Lausanne, Switzerland; 2 Bioinformatics Competence Center, Ecole Polytechnique Fédérale de Lausanne, 1015 Lausanne, Switzerland; 3 Bioinformatics Competence Center, University of Lausanne, 1015 Lausanne, Switzerland; 4 Swiss Institute of Bioinformatics (SIB), 1015 Lausanne, Switzerland; 5 Thermo Fisher Scientific, Parc Tertiaire Bois-Dieu, 69380 Lissieu, France; 6 Thermo Fisher Scientific, 2130 Woodward Street, Austin, TX 78744 USA

**Keywords:** Amplicon sequencing, Population management, Whitefish, *Coregonus*, Parentage assignment

## Abstract

Fish populations are notoriously difficult to monitor because their aquatic habitats limit observation. While physical or chemical tagging (e.g., adipose fin removal or Alizarin Red S staining of bones) can reduce growth or survival or requires sacrifice, genetic tools offer a less invasive and more informative alternative, allowing managers to assess stock composition and census size while enabling researchers to study ecological and evolutionary processes. Here, we introduce a novel whitefish (*Coregonus* spp.) amplicon panel with 2,282 SNP loci identified using whole-genome resequencing, targeting loci spread across the whitefish genome, including candidate regions relevant to growth and immunity. We successfully applied the panel in seven whitefish species (*C*. cf. *suidteri*, *C. confusus*, *C. albellus*, *C. muelleri*, *C. fatioi*, *C. palaea*, and one *C. sp.*) from seven Swiss lakes. To evaluate performance, we used offspring of known parentage from an in vitro breeding experiment with wild-caught *C. confusus*. Parentage assignment on 381 families yielded a 0% error rate, even when parental genotypes were missing. This panel provides a robust tool for a wide range of applications in broad taxonomic and geographic contexts for monitoring whitefish populations, a group that has historically been difficult to study within the salmonid family.

**Supplementary Information:**

The online version contains supplementary material available at https://doi.org/10.1007/s12686-026-01449-2.

## Introduction

Monitoring is (i) essential in the management of natural resources because it helps managers understand the consequences of management decisions, such as harvest, supplementary breeding, translocations, or genetic rescue, and (ii) has led to key insights into fundamental aspects of ecology and evolution. However, monitoring is logistically challenging, requiring significant investments in time, cost, and expertise. This is especially true for aquatic populations because sampling and observing species underwater is complex, and monitoring based on physical or chemical tagging is often stressful to the fish and can reduce growth or survival (McKenzie et al. 2012; Meyer et al. 2012; von Siebenthal et al. 2017).

Genetic markers offer a less invasive alternative, requiring only a small tissue sample, such as a small piece of a fin that quickly regrows, allowing individuals to be promptly released with fewer long-term negative impacts. Due to their high polymorphism and relatively low sequencing cost, microsatellites were among the first widely adopted markers and remain the genetic marker of choice for managing many fish populations. They have been used for a wide range of applications including population estimation (Miller et al. 2015; Bravington et al. 2016a; Marcy-Quay et al. 2020; Prystupa et al. 2021), investigation of genetic structure (Stelkens et al. 2012; Layton et al. 2020; Lehnert et al. 2020), stock identification (Bradbury et al. 2018; Layton et al. 2020; Marcy-Quay et al. 2023), differentiating between natural-born and stocked fish (DeHaan et al. 2008; Hsu et al. 2020; Palejowski et al. 2022; Hargrove et al. 2022; Fitzpatrick et al. 2023), testing the effects of crossing genetically distinct populations on embryo survival (Stelkens et al. 2014), determining dispersal and philopatric propensity (Salles et al. 2016; Rueger et al. 2020), as well as investigations of how sex, genetic, and environmental factors early in life influence growth of individuals in the wild (Bylemans et al. 2024a, b).

Decreasing sequencing costs have increased adoption of other genetic markers, such as single-nucleotide polymorphisms (SNPs) for population monitoring. SNPs are abundant in the genome, have low sequencing error rates, and can occur in functional regions, making them versatile for a broad range of applications (Flanagan and Jones 2019; Meek and Larson 2019). In the context of monitoring fish populations, SNPs have been used for stock assignment (Steele et al. 2013; Hess et al. 2016), to investigate dispersal (Schunter et al. 2014), to quantify introgression between wild and farm raised fish (Karlsson et al. 2016), to reconstruct pedigrees for breeding programs (Xu et al. 2017; Holman et al. 2017), for broodstock screening (Lew et al. 2015), and to understand important life history traits such as the timing of migration and spawning (Abadía-Cardoso et al. 2013; Hess et al. 2016).

Targeted amplicon sequencing provides an exciting opportunity to develop panels of genetic markers for monitoring wild populations because it is specific, enabling genotyping at high depth at low cost, making population-wide assessments feasible (Aykanat et al. 2016; Meek and Larson 2019; Bootsma et al. 2020). Additionally, amplicon markers can be designed to be neutral or to target functional regions of the genome, enabling a wide range of applications. Amplicon sequencing can target diverse marker types within a single panel, including microsatellites, SNPs, or whole genes, enabling creative monitoring approaches that combine the distinct benefits of each marker. Recent creative uses of amplicon sequencing in conservation and management include sequencing amplicons from fecal samples of cryptic species (Wolfenson et al. 2024) and retrofitting legacy markers that are part of long-term monitoring programs (Gruenthal and Larson 2021). In the context of fish monitoring, amplicon sequencing has been used to investigate changes in demography before and after spawning (Matala et al. 2016), for mixed stock analysis using parentage assignment (Beacham et al. 2017, 2018, 2019; Anderson et al. 2025), and even the retrofitting of loci from a panel designed for walleye (*Sander vitreus*) for use in differentiating from, and management of, the closely related but less studied sauger (*Sander canadensis*) (Homola et al. 2024).

Alpine whitefish (*Coregonus* spp.) represent a challenging group for fisheries management compared to other species (Douglas and Brunner 2002). Whitefish have a history of being heavily impacted by anthropogenic stressors, most notably overfishing (Smith 1964; Weidel et al. 2022; Bunnell et al. 2023, 2024) and eutrophication (Vonlanthen et al. 2012; Bunnell et al. 2024), which has resulted in loss of genetic diversity (Hudson et al. 2010; Frei et al. 2024), local extirpations (Bunnell et al. 2023), species extinctions (Freyhof and Schöter 2005; Vonlanthen et al. 2012; Frei et al. 2023; Bunnell et al. 2023), as well as introgression between species and even speciation reversals (Vonlanthen et al. 2012; Feulner and Seehausen 2019; Frei et al. 2022, 2023). Their long history of exploitation has led to extensive management, including numerous translocations (Huuskonen et al. 2004; Doenz et al. 2018; Bunnell et al. 2024) and supplemental breeding programs (Dierking et al. 2014), which likely led to artificially induced introgression between species. Given the costs and risks of negative impacts, there is great interest in tools that can be used to quantify the efficacy of whitefish supplemental breeding programs on targeted stocks, as some studies found significant impacts on wild populations (Anneville et al. 2009; Wanke et al. 2016; Weidel et al. 2022; Wedekind et al. 2022), while others found little to no effects (Baer et al. 2023). Microsatellites have long been used in whitefish management and research (e.g. Douglas and Brunner 2002; Winkler et al. 2011; Hudson et al. 2017; Doenz et al. 2018). Shi et al. (2022), working on whitefish of Lake Michigan, demonstrated that denser genomic tools that include adaptive markers can provide more detailed insights.

Here we present an amplicon panel containing 2,282 biallelic SNP loci designed via whole genome re-sequencing of whitefish (*Coregonus* cf. *suidteri)* from Lake Hallwil, containing loci spread across the genome, including candidate loci associated with growth and immunity. We validate this panel for use in managing whitefish (*Coregonus* spp.) using new samples from Lake Hallwil as well as an additional 6 species of whitefish (*C. confusus*,* C. albellus*,* C. muelleri*,* C. fatioi*, *C. palaea* and one undescribed *C. species*) from a further 6 Swiss lakes (Biel, Brienz, Geneva, Joux, Lucerne, and Thun) by testing per locus variability and callability. Lastly, we ensure the panel can differentiate hatchery-raised whitefish from their wild counterparts via parentage assignment using singly-reared larvae from a 20 × 20 full-factorial breeding experiment with wild-caught *C. confusus*.

## Materials and methods

### Whole genome re-sequencing and amplicon design

#### Sampling, DNA extraction, and library preparation

Thirty-four male and twenty female whitefish (*C.* cf. *suidteri*) from Lake Hallwil were collected by gillnet fishing during two spawning seasons, December 2016 to January 2017 (34 males, 3 females), and December 2020 (17 females). Tissue samples were taken from the anal fin and stored in 70% ethanol at −20° C. Samples were processed with a DNeasy blood & tissue kit (Qiagen, Hilden, Germany). DNA integrity was checked via electrophoresis on a 1% agarose gel, and final concentration was determined using a Qubit dsDNA HS assay kit (*ThermoFisher*, Waltham, Massachusetts, United states).

DNA from the individuals collected during the 2016–2017 breeding season was used to prepare TruSeq PCR-free libraries (Illumina, San Diego, California, United States) according to the manufacturer’s instructions for a DNA insert size of 550 bp. Library quality was assessed using a KAPA library quantification kit (Roche Diagnostics Corp, Basel, Switzerland) in conjunction with a fragment analyzer (Advanced Analytical Technologies, Fiorenzuola d’Arda, Italy). Libraries were then sequenced on the Illumina HiSeq 4000 platform (Illumina, San Diego, California, United States) at the Lausanne Genomic Technologies Facility. Additionally, the 17 individuals collected during the 2020 breeding season were used to prepare TruSeq Nano DNA libraries (Illumina, San Diego, California, United States) according to the manufacturer’s instructions for a DNA insert size of 550 bp. Library quality and concentrations were assessed using a fragment analyzer and qPCR with a *LightCycler* 480 (Roche Diagnostics Corp, Basel, Switzerland). Individuals were then sequenced on the Illumina NovaSeq 6000 platform (Illumina, San Diego, California, United States) at the Montpellier GenomiX center.

#### Resequencing

All steps of resequencing including quality controls and variant calling were performed across sampling periods (i.e. on all 54 individuals together). *RepeatModeler* (v.2.0) (Flynn et al. 2020) was used for *de novo* repeat identification in the *Coregonus* cf. *suidteri* genome (GenBank accenssion GCA_036851935.1). *RepeatMasker* (v.4.1.0) (Smit et al. 2015) was subsequently used to annotate repetitive DNA sequences in the genome. To ensure the inclusion of bases at the extremities, a custom bash script was used to adjust the repeat-masked intervals by decrementing the start positions by 1 and incrementing the end positions by 1. Finally, unmasked regions were extracted using *bedtools complement* (v2.29.2) (Quinlan 2014). The whole-genome re-sequencing data were preprocessed using *Trimmomatic* (Bolger et al. 2014). Adapter sequences were detected and removed. Quality trimming was applied to both ends of the reads (LEADING and TRAILING with a quality threshold of 3) to eliminate low-quality bases. Reads shorter than 70 bases after trimming were discarded to ensure the dataset comprised only high-quality, informative sequences. The resulting reads were aligned to the whitefish Coregonus cf. suidteri genome using the BWA-MEM algorithm in the *BWA* (v.0.7.17) software package (Li 2013). Post-alignment, *SAMtools* (v.1.12) (Li et al. 2009; Danecek et al. 2021) was used to sort the alignments. *Picard* (v.2.24.0) was applied to add read group information (*AddOrReplaceReadGroups*), and *CleanSam* was used for soft-clipping alignments extending beyond scaffold boundaries. After sorting, pairing, and removing secondary alignments, *Picard* was used to identify and flag duplicate sequences (*MarkDuplicates*), generate a sequence dictionary, and reorder BAM files according to the reference genome ordering. Finally, high-quality primary alignments were filtered via *SAMtools view* (-F 256, -q 60) and indexed before undergoing comprehensive quality control with *Qualimap2* (Okonechnikov et al. 2016). Variant calling was conducted using *GATK* (v.4.2.0.0) *HaplotypeCaller* (Van der Auwera & O’Connor 2020). Post-genotyping, variants were filtered via *VariantFiltration: QD* < 2, *QUAL* < 30.0, *SOR* > 3, *FS* > 60.0, *MQ* < 40.0, *MQRank* < −12.5, and *ReadPosRankSum* < −8. These filters reduce the chance of false positives, enhancing the quality of the loci for downstream analysis. Next, *VCFtools* (v.0.1.14) (Danecek et al. 2011) was used to restrict the dataset to biallelic single-nucleotide polymorphisms (SNPs), omitting indels and multiallelic sites. Variants were further refined by filtering out individual genotypes with a genotype quality (*GQ*) score below 30, discarding sites with more that 10% missing genotypes, and coverage to a minimum of 10X and a maximum of 50X. To remove problematic markers resulting from null alleles or paralog-specific amplification —a common concern in recently diverged, duplication-rich salmonid genomes—markers deviating from Hardy-Weinberg equilibrium were excluded with *VCFtools*’ two-tailed exact test using a uncorrected threshold of *p* < 0.05. Variants exhibiting excessive heterozygosity (*ExcessHet* > 10.10 in *GATK*) were removed, and only SNPs with a minor allele frequency (MAF) of 0.3 or higher were retained. Although these filters for Hardy-Weinberg and heterozygosity along with the samples from two spawning seasons being pooled could lead to filtering out not just problematic variants, but also variants that are out of equilibrium due to selection or from family or population structure, the risks of this in the context of this study are low. The whitefish population in Lake Hallwil is large with 1,000s to 10,000s of individuals harvested each year, making family structure among sampled individuals unlikely. Additionally, unlike other Alpine lakes, Lake Hallwil does not host sympatric whitefish ecotypes *(*Wedekind et al. 2022), and has a documented history of eutrophication which is known to have reduced whitefish population structure in Swiss lakes *(*Vonlanthen et al. 2012). Finally, linkage disequilibrium (LD) pruning was performed using the *indep-pairwise* function in *PLINK* (v.1.9) (Purcell et al. 2007; Chang et al. 2015) with sliding windows of 50 SNPs, a step size of 5 SNPs, and an r2 threshold of 0.25 to filter out closely linked variants. Subsequently, *bcftools* (Danecek et al. 2021) was used to extract variants from this pruned dataset corresponding exclusively to the unmasked genomic regions, resulting in a VCF file containing only variants from these specified regions. The final VCF was compressed using *bgzip* and indexed via *bcftools* index.

#### SNP selection for amplicon design

To facilitate long-term monitoring of whitefish-specific regions of the *C.* cf. *suidteri* genome, a panel was designed that included loci spread across the genome, as well as candidate loci associated with immunity and growth. *BLAST* (Altschul et al. 1990) was used to align Atlantic salmon (*Salmo salar*, genome, GenBank ascension GCA_905237065.2), coho salmon (*Oncorhynchus kisutch*, genome GenBank ascension GCA_002021735.2), and sockeye salmon (*Oncorhynchus nerka*, genome GenBank ascension GCA_006149115.1) reference sequences to find homologous regions in the whitefish genome that correspond to genes associated with immunity and/or growth. Candidate regions related to immunity included 4 MHC class I alpha regions, the MHC class I beta region, the MHC class II alpha and beta chains, and the regulatory factor 1 (RFX1), these regions were chosen as MHC genes are well known to influence immunity across teleosts, and are well described in salmonids (Wedekind et al. 2004; Grimholt 2016).

Candidate genes related to growth included vestigial-like family member 3 (VGLL3), ectodysplasin A receptor (EDAR), Insulin-like growth factor 1 (IGFI), growth hormone 1 (GH1) and 2 (GH2), and keratin types 1 (KT1) and 2 (KT2), all of which are known to influence growth in salmonids or other teleost’s *(*Yamamoto et al. 2016; Kusche et al. 2017; He et al. 2020; Triantaphyllopoulos et al. 2020; De-Kayne et al. 2022).

Locus selection focused on the first 50 scaffolds of the assembly, targeting 826 SNPs within the candidate gene regions alongside an additional 2,000 randomly selected SNPs. These 2,000 loci, distributed across the targeted scaffolds, and were filtered to conform to Hardy-Weinberg expectations. As they were randomly sampled outside of any candidate genes, we refer to them as putatively neutral SNPs hereafter. To facilitate primer design, the precise positioning of all selected variants was validated using *Minimap2* (Li 2018) and *bedtools getfasta* was used to extract the 200 bp flanking sequences on both sides of each SNP, resulting in 401 bp sequences. Sequence information for 2,000 putatively neutral, 424 growth-associated, and 402 immunity-associated loci was then sent to *ThermoFisher* in Lissieu, France, for primer design.

#### Primer design

The list of submitted markers was passed through *ThermoFisher’s* quality control process to verify their correctness against the whitefish reference genome. Markers that failed quality control due to multimapping, incorrect positions, primer-dimer interactions, or duplication were removed. Primers were then designed for the markers that passed through quality control using the AgriSeq primer design pipeline.

### Panel validation

#### Panel performance across whitefish species

For panel validation, an additional 227 whitefish representing 7 species that had been sampled from 7 lakes between 2003 and 2022 were used. These species include *C. confusus* (Lake Biel), *C. albellus* (Lake Brienz), *C. palaea* (Lake Geneva), *C. sp. (*Lake Joux*)*,* C. mulleri* (Lake Lucerne), *C.* cf. *suidteri* (Lake Hallwil), and *C. fatioi* (Lake Thun), with sample size per lake ranging from 14 to 93 individuals (Table 1). Tissue samples (anal fin, adipose fin, or liver tissue) had been taken and placed in 70% ETOH (anal fin, adipose fin, liver tissue) or dried (anal fin) prior to long-term storage (Table 1). DNA from these samples was extracted using the same methodology outlined above, except that in the case of dried tissue, they were first ground to a powder with a mortar and pestle before extraction. All samples were quantified using a DeNovix™ DS-11 series Spectrophotometer/Fluorometer (DeNovix Inc, Delaware) to ensure they had a minimum quantity of 10 ng/µl. Prior to sequencing, samples were normalized to a final concentration of 3.3 ng/µL. The individuals from lake Biel were used as technical replicates, undergoing two separate rounds of extraction, library prep, and sequencing. Libraries were constructed with an AgriSeq™ HTS library kit (*ThermoFisher*, Waltham, Massachusetts, United States) following the protocol for 96-well plates, 14 8-minute amplification cycles, and two rounds of magnetic bead-based cleanup to remove residual reagents, and to size select the library and exclude smaller fragments such as primer dimers. Lastly, libraries were sequenced by *ThermoFisher* in Lissieu, France, on their Ion Torrent NGS platform using the Ion 550™ chip and Ion 550™ kit - Chef. A minimum coverage depth of 20x was used, with any individuals below 20x at a given locus being unscored at that locus.

**Table 1:** Sample details for testing panel performance across different whitefish species

Lake	Species	*N* (extracted)	*N* (after filtering)	Retention rate	Sample type	Storage conditions (° C)	Year of sampling
Biel	*C. confusus*	40	40	100%	Anal fin clip in 70% ETOH	−20°	2022
Brienz	*C. albellus*	16	15	93.75%	Adipose fin in 70% ETOH	Room temperature	2008
Geneva	*C. palaea*	14	11	78.57%	Liver in 70% ETOH	Room temperature	2005
Hallwil	*C.* cf. *suidteri*	93	92	98.93%	Anal fin clip in 70% ETOH	−20°	2019
Joux	*C.* sp.	16	10	62.5%	Adipose fin in 70% ETOH	6.5°	2007
Lucerne	*C. muelleri*	16	15	93.75%	Dried fin	Room temperature	2003
Thun	*C. fatioi*	16	16	100%	Dried fin	Room temperature	2004
Thun	*C. fatioi*	16	16	100%	Adipose fin in 70% ETOH	6.5°	2004

Retention rate refers to the percentage of individuals remaining after quality filtering, see main text for filtering criteria

The raw data generated after sequencing were processed using the *Torrent Suite*™ Software for signal processing, base calling, read filtering, and demultiplexing reads into individual samples based on barcode sequences. For each sample, the reads were mapped to the whitefish reference genome using the *Torrent Mapping Alignment Program TMAP* (https://github.com/iontorrent/TMAP), followed by genotyping using *TVC-Torrent Variant Caller* (https://github.com/iontorrent/Torrent-Variant-Caller-stable). The *AgriSum Toolkit* plugin was used for quality checking. Poor-performing samples (i.e., individuals with low coverage) were re-sequenced by preparing new libraries with 3 additional amplification cycles.

Following sequencing, individuals who were scored for < 80% of all loci were removed from further analyses. Per locus reliability was determined using the *SnpStats()* function in the R package *SEQUOIA* (3.0.3) (Huisman 2017) by comparing discordance in genotype calls between the individuals from lake Biel, which were used as technical replicates. Locus variability and callability were determined by calculating allele frequencies per lake and locus, as well as the proportion of individuals genotyped per lake and locus, using *Genodive* (Meirmans 2020). A locus was considered callable if it was scored for ≥ 80% of individuals in a given lake. It was considered highly variable if it had a reference allele frequency between 0.3 and 0.7 in a given lake.

#### Experimental breeding block and parentage assignment

In order to validate the panel for parentage assignment, gametes from the whitefish collected from Lake Biel (*C. confusus*) were crossed in vitro in a large 20 × 20 full-factorial breeding design to produce 400 unique families. The milt from each of the 20 males was used to fertilize the eggs from each of the 20 females (separately) in Petri dishes in all possible combinations for this and a parallel study (Beck et al. in preparation). Fertilized eggs were left to harden for two hours before being transported on ice to a climate-controlled room set at 6.5 °C. The embryos were then washed and distributed singly into individual wells filled with 2 mL of standardized water, following the protocol of Clark et al. (2014). Embryos were allowed to develop to at least the eyed stage, then sampled and stored in 70% ethanol at −20 °C prior to DNA extraction. At the time of sampling, 381 of the original 400 families had surviving offspring. Therefore, one offspring per family of the 381 remaining families was used to assess the error rates of parentage and half-sib assignment. The DNA of the offspring was extracted, quantified, stored, and sequenced following the same process outlined above for the adult whitefish collected from each of the seven lakes.

The parental assignment was performed using both *COLONY* (2.0.6.8) (Jones and Wang 2010) and *SEQUOIA*. *COLONY* was chosen due to its high prevalence in the literature and its MCMC likelihood approach, which is known to be powerful albeit computationally intensive. *SEQUOIA* was chosen because it uses a heuristic hill climbing algorithm that implements a filtering step early on by omitting individuals who are unlikely to be related due to alternative homozygous genotypes, resulting in a less computationally intensive analysis and, therefore, a quicker analysis compared to *COLONY*.

To investigate rates of incorrect parentage assignment, the parents and offspring from the full-factorial breeding design underwent two rounds of parentage assignment in both *SEQUOIA* and *COLONY*. For parentage assignment, only putatively neutral loci that were genotyped in > 80% of individuals and had a reference allele frequency between 0.25 and 0.75 were used, and individuals were checked to ensure that they were missing < 10% of genotypes, as missing data or markers with low variability are known to lead to errors in parentage assignment. One run was performed on each software which contained the genotypes of all parents and offspring. However, in real-world scenarios the genotypes of all parents may not be available. Therefore, a second run was performed in each software, in which ½ of parental genotypes were removed in order to test the rate of false-positive assignments (offspring assigned a parent whose genotype is not included in the dataset). Following this reduction the second run contained 98 offspring that had both parents genotypes present in the dataset, 196 offspring where only one of the parents genotypes remained (100 with only the sire, and 96 with just the dam respectively), and 87 offspring with neither parental genotype present. Both *COLONY* runs were done using the following settings: male and female polygamy, with inbreeding, without clones, dioecious, diploid, medium run length, full-likelihood analysis, medium precision, no sibship scaling, no prior sibship, no known maternal or paternal siblings, no excluded paternity, maternity, or siblingship, allelic dropout, and genotyping errors of 0.05. Both *SEQUOIA* runs were done with non-overlapping generations, and an error rate of 0.05. Additionally, the number of Mendelian errors observed among known parents and offspring was calculated using the *SnpStat()* function in *SEQUOIA* as an additional estimate of per locus reliability.

## Results

### Whole genome and amplicon panel

A total of 2,826 sequences were sent to *ThermoFisher* for primer design, each for a single biallelic SNP locus. Of these 2,826 loci, 2,000 were putatively neutral, 424 were candidate growth-associated, and 402 were candidate immunity-associated. 2,472 of the 2,826 markers passed *ThermoFisher’s* quality control, and for the final panel, primers were designed for 2,282 markers spread across 1,945 amplicons which ranged in length between 64 and 190 base pairs (average of 154.7). Of these markers, 1,755 are putatively neutral, each on its own amplicon. Since candidate gene-associated markers were understandably located closely to one another when compared to the putatively neutral markers, some are present on the same amplicon. In total, of the 2,282 markers 274 spread across 118 amplicons are candidate growth-associated. 74 of these amplicons carry more than one marker with an average of 2.32 per amplicon. Additionally, 253 of these markers spread across 72 amplicons are candidate immunity-associated amplicons with an average of 3.51 per amplicon.

**Fig. 1:**
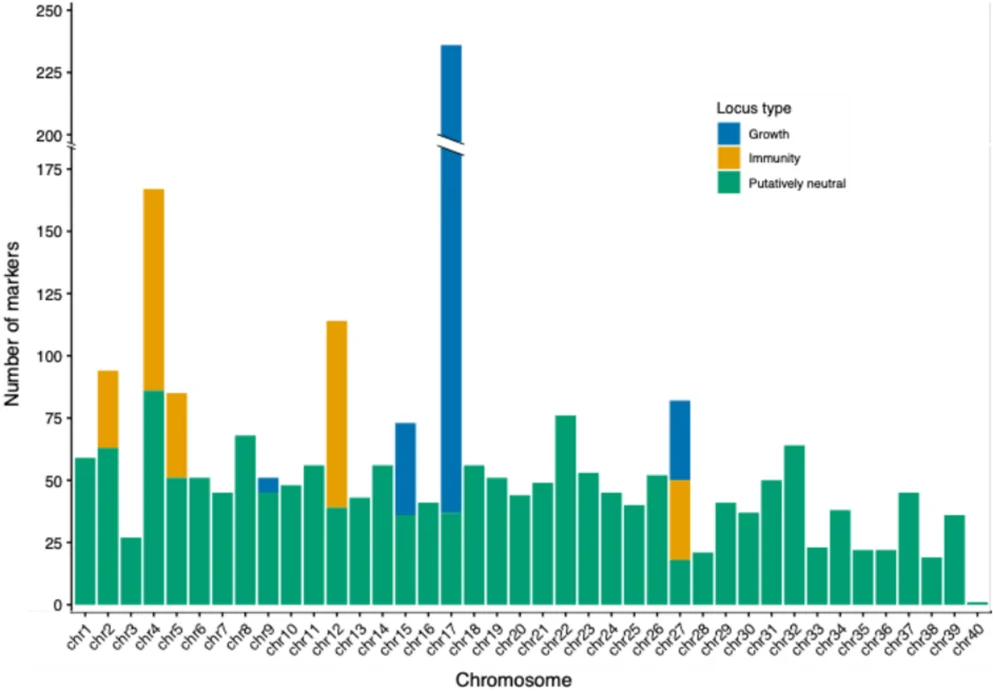
Count of putatively neutral, candidate growth, and candidate immunity loci per chromosome

The panel contains loci on 40 chromosomes as well as one locus on the unmapped scaffold 48. The average number of loci per chromosome was ~ 57, and ranged from 1 on chromosome 40 and scaffold 48, to 236 on chromosome 17 (Fig. 1). Putatively neutral loci were present on all chromosomes with an average of ~ 44 per chromosome and ranged from 1 locus (on chromosome 40 and scaffold 48 respectively) to 86 on chromosome 4. Candidate growth-associated loci were located on four chromosomes (9, 15, 17, and 27), with an average of 69 loci across these four chromosomes, and a range of 6 to 199. Lastly, candidate immunity-associated loci were located on five chromosomes (2, 4, 5, 12, and 27) with an average of ~ 51 loci across these five chromosomes with a range from 31 to 81.

### Amplification rate, loci reliability, callability, and variability

Following sequencing, 12 of 227 individuals were removed from further analysis for having been genotyped at < 80% of loci. This includes 6 individuals from Lake Joux, 3 individuals from Lake Geneva, and one individual from Lakes Lucerne, Brienz, and Hallwil, each. Table 1 gives the remaining sample size from each lake post-filtering. Per-locus genotyping error was low: estimation of genotyping error rates yielded an average discordance rate of 2.44% between technical replicates from Lake Biel, and on average a 2.11% rate of Mendelian errors among known parent-offspring trios.

**Fig. 2:**
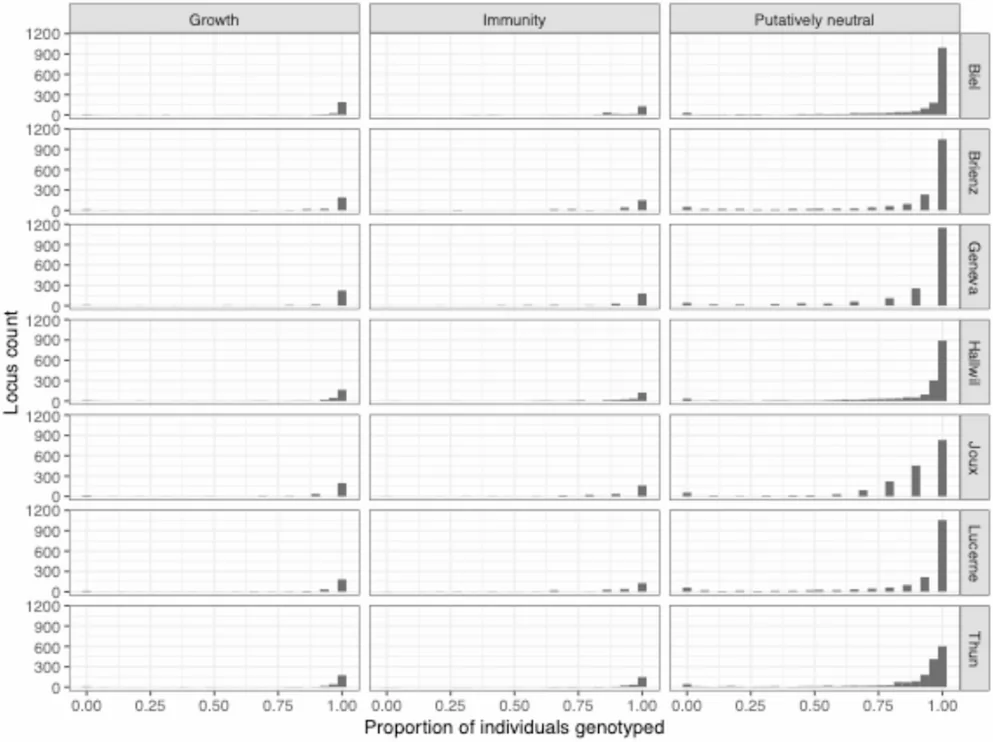
Count of putatively neutral (*N* = 1,755), candidate growth (*N* = 274), and candidate immunity (*N* = 253) loci where a given proportion of individuals were genotyped in whitefish from 7 Swiss lakes

The proportion of callable loci (loci scored in ≥ 80% of individuals in a given lake) was high and consistent across all three locus types across all lakes (Fig. 2), with the number of callable candidate growth-associated loci ranging from 235 in Lake Lucerne to 245 in Lake Hallwil, while the number of callable candidate immunity-associated loci ranged from 204 in Lakes Brienz and Lucerne to 223 in Lakes Biel and Joux. Lastly, the number of callable putatively neutral loci ranged from 1407 in Lake Geneva to 1512 in Lake Joux.

**Fig. 3:**
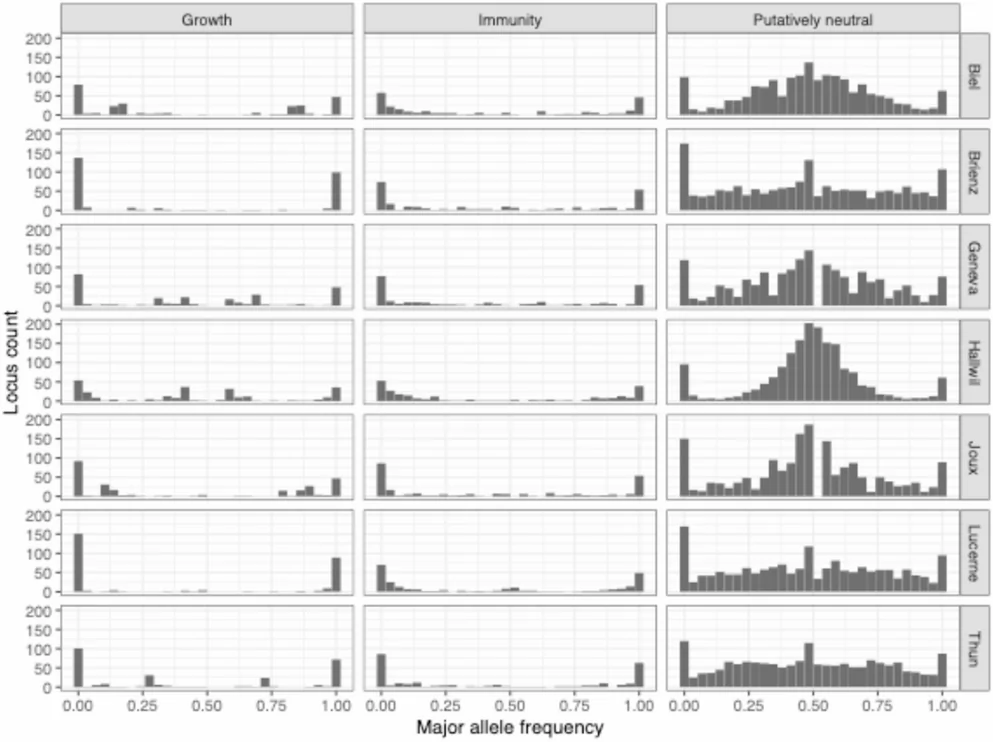
Count of putatively neutral (*N* = 1,755), candidate growth (*N* = 274), and candidate immunity (*N* = 253) loci with a given reference allele frequency in whitefish from 7 Swiss lakes

 The number of candidate growth-associated loci that were highly variable ranged from 10 in Lakes Joux and Lucerne to 121 in Lake Hallwil. The number of candidate immunity-associated loci that were highly variable ranged from 18 in Lake Thun to 47 in Lake Brienz. Lastly the number of putatively neutral loci that were highly variable ranged from 730 in Lakes Thun and Brienz to 1,359 in Lake Hallwil (Fig. 3, Table S1).

### Parental assignment

After sequencing and filtering, a total of 1,128 putatively neutral loci were retained for parentage assignment. Individuals ranged from having 90–99.5% of genotype data, so all individuals were retained for further analysis. Results from all parentage assignment runs were 100% correct with no false positives nor false negatives in either *COLONY* or *SEQUOIA*. In the first run where all parental genotypes were included, all offspring were assigned to their correct parents. In the second run where ½ of the parental genotypes were removed, the 98 offspring that had both parents remaining in the dataset were correctly assigned to their corresponding parents, the 196 individuals with only one parent remaining in the dataset were assigned to that parent only, and the 87 offspring that had neither parent remaining in the dataset were correctly left with no parents assigned. No full siblings were included in our experimental design to explore full-sib assignment. However, among our 381 offspring there was a total of 6,915 half-sib dyads that could be used to evaluate half-sibling assignments. All half-sibling relationships were correct across both runs of *SEQUOIA*, with no incorrect full-sibling assignments, no false positives or false negatives. In the second *SEQUOIA* run where only ½ of the parental genotypes were included, dummy parent(s) were assigned as placeholders. When one or both of the parents was not present, all half-siblings shared the correct parent (if it was included in the dataset) or the correct dummy parent(s) if their parent(s) were not included. No false full-sibling assignments were made across either run of *COLONY.* In the first *COLONY* run with all parental genotypes all 6,915 half-sibling dyads were correctly assigned. In the second *COLONY* run, *COLONY* correctly identified 6,891 of the 6,915 half-sibling dyads (99.7%), 6,715 (97%) had a probability of 0.9 or greater, 176 (2.5%) of the dyads had a probability below 0.9, and 24 (0.3%) of dyads were not identified (i.e. were false negatives).

## Discussion

We present a whitefish (*Coregonus* spp.) amplicon panel comprising 2,282 SNP loci distributed throughout the whitefish genome, some in candidate regions associated with growth or immunity. We tested this panel on 7 whitefish species from 7 Swiss lakes, all of which are targeted by commercial and recreational fishing. Our analyses revealed that the panel contains hundreds of variable and callable loci across all species and lakes. Additionally, we used a large full-factorial in vitro breeding experiment and single rearing of larvae to validate that the panel is suitable for reliable parentage assignment, which can be used to differentiate hatchery-reared individuals from their wild-born counterparts (DeHaan et al. 2008; Hsu et al. 2020; Palejowski et al. 2022; Hargrove et al. 2022; Fitzpatrick et al. 2023).

The parentage analysis from our 20 × 20 full-factorial breeding design demonstrated error-free parentage assignment in all 381 experimental families: offspring were correctly assigned to both parents when both genotypes were present, to the single available parent when only one was present, and left unassigned when neither was present. Additionally, half-sibship construction yielded either no (in both runs using *SEQUOIA*, and the run in *COLONY* with half the parental genotypes included), or low (97% correct half-sibling dyad reconstruction with 0.9 or greater probability, 0.3% false negative error rate, no false positives) rates of error in the *COLONY* run with half the parental genotypes missing.

Multi-species panels are useful because they provide managers with a single tool that can be used across broad geographic and taxonomic contexts. Other multi-species amplicon panels have been designed for different purposes, such as assessment of genetic diversity in multiple plant species in temperate grasslands (Loera-Sánchez et al. 2022), to identify snail species as well as their trematode parasites (Hammoud et al. 2022), monitoring of malaria vectors (Makunin et al. 2022), and to differentiate between the closely related walleye (*Sander vitreus*) and sauger (*Sander canadensis*) (Homola et al. 2024). Although our panel was shown to contain many callable and variable loci in all 7 whitefish species investigated, caution is warranted regarding the multi-species applications of the panel, as designing markers that would allow to distinguish between species similar to Hammound et al. (2022), or Homola et al. (2024), was outside the scope of this study (see supplementary materials for further details on genetic structure analysis among our samples).

The panel we present here was not intended to allow for distinguishing between whitefish species. For whitefish, designing such a tool is challenging for many reasons. *Coregonus* is a species-rich genus, comprising over 65 species distributed across the Northern Hemisphere (Kottelat and Freyhof 2007; Eshenroder et al. 2016). Whitefish species diversity is the result of two notable radiations, one in the Laurentian Great lakes of North America, and, another in the peri-Alpine lakes of Europe, which are estimated to have started during periods of de-glaciation ~ 80kya (Backenstose et al. 2024) and ~ 15kya (Østbye et al. 2005) respectively. In the peri-Alpine region, the rapid colonization of post-glacial lakes has resulted in the emergence of many whitefish species that live in sympatry, with as many as six species in some lakes (Kottelat and Freyhof 2007; Hudson et al. 2010; Doenz et al. 2018), with multiple allopatric species that occupy similar niches and share similar morphology (Kottelat and Freyhof 2007; Doenz et al. 2018). Peri-Alpine whitefish are known to represent species flocks, meaning that species in sympatry are more closely related to one another than to allopatric species (Douglas et al. 1999). As a result, there has been a long history of disputes regarding the taxonomic delineations of whitefish. Although there is a recent and ongoing effort to establish clear delineations between species (Selz et al. 2020; Selz and Seehausen 2023), distinguishing between sympatric species based on morphology remains difficult for managers, and the close relatedness of sympatric species makes developing genetic tools to distinguish them challenging. However, with decreasing sequencing costs, the development of markers to distinguish morphologically and genetically similar species living in sympatry represents a natural next step and would represent a significant leap forward in the ongoing management of this species-rich genus.

The high variability and callability of our loci across the 7 sampled species, along with the high rates of correct parentage and half-sib assignment, demonstrate that this panel would be applicable to species across the peri-Alpine region and perhaps beyond. This panel could be used for a variety of applications including: assessing genetic diversity (Stelkens et al. 2012; Bradbury et al. 2018; Layton et al. 2020; Lehnert et al. 2020; Marcy-Quay et al. 2023), estimation of population abundance via close-kin mark-recapture (Bravington et al. 2016b; Waples and Feutry 2022; Casas and Saborido-Rey 2023a, b; Babyn et al. 2024), assessing genetic and phenotypic differentiation, and hybridization between hatchery-reared and wild-born individuals using parental assignment (Stelkens et al. 2014; Hess et al. 2016; Janowitz-Koch et al. 2019; O’Sullivan et al. 2020; Palejowski et al. 2022), or investigation of rates of philopatry and dispersal by estimating relatedness and/or pedigree construction among individuals (Waser et al. 2006; Schunter et al. 2014; Salles et al. 2016; Scott et al. 2018; Rueger et al. 2020). Together, these uses and the panel’s broad geographic and taxonomic applicity make it a powerful tool for whitefish population management going forward.

## Supplementary Information

Below is the link to the electronic supplementary material.

Supplementary Material 1

## Data Availability

The chromosome-scale genome assembly for the whitefish, *Coregonus* cf. *suidteri* can be found on the European Nucleotide Archive with the accession GCA_036851935. Raw sequencing files for the re-sequenced *Coregonus* cf. *suidteri* individuals can also be found at the European Nucleotide Archive under study ascensions PRJNA715057, and PRJEB111942. All other data generated and analyzed during this project, including marker sequences, raw amplicon sequencing files, parentage assignment input/output, and scripts have been uploaded to Dryad DOI: https://doi.org/10.5061/dryad.73n5tb3cj.
