# Supplementary material for: A validated amplicon panel for parentage reconstruction and selection monitoring in whitefish (Coregonus spp.)
Source: Conserv Genet Resour. 2026 Oct 16;18(4):43. doi: 10.1007/s12686-026-01449-2 (PMC13675297; doi:10.1007/s12686-026-01449-2)
Supplement: Supplementary file 1 [file 12686_2026_1449_MOESM1_ESM.docx]

**Supplementary material**

### A validated amplicon panel for parentage reconstruction and selection monitoring in whitefish (*Coregonus* spp.)

Caleb Beck^1,A^ , Marion Leleu^2,3,B^, Marina Braso Vives^1,4,C^, Stephane Daly^5^, Sandesh Shrestha^6^, Gregory Brazzola^1^, Claus Wedekind^1,D^

^1^ Department of Ecology and Evolution, University of Lausanne, 1015 Lausanne, Switzerland

^2^ Bioinformatics Competence Center, Ecole Polytechnique Fédérale de Lausanne, 1015 Lausanne, Switzerland

^3^ Bioinformatics Competence Center, University of Lausanne, 1015 Lausanne, Switzerland

^4^ Swiss Institute of Bioinformatics (SIB), 1015 Lausanne, Switzerland

^5^ Thermo Fisher Scientific, Parc Tertiaire Bois-Dieu, 69380 Lissieu, France

^6^ Thermo Fisher Scientific, 2130 Woodward Street, Austin, TX 78744, USA

^A^ Orchid ID: 0009-0007-3060-6774

^B^ Orchid ID: 0000-0002-1800-0821

^C^ Orchid ID: 0000-0001-5007-5789

^D^ Orchid ID: 0000-0001-6143-4716

^D^ corresponding author: [claus.wedekind@unil.ch](mailto:claus.wedekind@unil.ch)

**Content**

Table S1: Number of loci associated with growth, immunity, and putatively neutral loci.

Table S2: Between-lake *F_ST_* values of whitefish from 7 Swiss lakes

Figure S1: Optimal number of clusters (Delta K) of the 7 whitefish species.

Figure S2: Genetic structure of the 7 whitefish species

Table S1: Number of callable and variable loci associated with candidate growth, candidate immunity, and putatively neutral loci.

| Lake | Loci with the proportion of individuals genotyped at ≥0.8 | | | | Loci with the frequency of the reference allele at 0.3-0.7 | | | |
| --- | --- | --- | --- | --- | --- | --- | --- | --- |
|  | Growth | Immunity | Putatively neutral | Growth | | Immunity | Putatively neutral |  |
| Biel | 237 | 223 | 1431 | 14 | | 39 | 1070 |  |
| Brienz | 241 | 204 | 1444 | 14 | | 47 | 730 |  |
| Geneva | 241 | 213 | 1407 | 116 | | 39 | 953 |  |
| Hallwil | 245 | 217 | 1452 | 121 | | 22 | 1359 |  |
| Joux | 240 | 223 | 1512 | 10 | | 41 | 1061 |  |
| Lucerne | 235 | 204 | 1439 | 10 | | 38 | 764 |  |
| Thun | 243 | 222 | 1437 | 17 | | 18 | 730 |  |

Table S2: Between-lake *F_ST_* values of whitefish from 7 Swiss lakes, calculated using 1430 neutral SNPs with a minimum call rate of 0.8.

|  | Brienz | Geneva | Hallwil | Joux | Lucerne | Thun |
| --- | --- | --- | --- | --- | --- | --- |
| Biel | 0.104 | 0.026 | 0.052 | 0.026 | 0.121 | 0.093 |
| Brienz |  | 0.136 | 0.117 | 0.143 | 0.155 | 0.129 |
| Geneva |  |  | 0.061 | 0.020 | 0.144 | 0.115 |
| Hallwil |  |  |  | 0.055 | 0.106 | 0.101 |
| Joux |  |  |  |  | 0.156 | 0.120 |
| Lucerne |  |  |  |  |  | 0.173 |

Figure S1: Optimal number of clusters (Delta K) of 7 whitefish species, based on analysis run in *Structure* (Pritchard et al. 2000; Falush et al. 2003) with a burn-in length of 10,000, followed by 100,000 MCMC replications, using the admixture model, sampling locations as a prior, and correlated allele frequencies. K was estimated from 1-7, and the probability of the data for estimating K was computed in order to identify Delta K (Evanno et al. 2005). This run included the 215 individuals from the 7 Swiss lakes who remained after sequencing and quality filtering (Table 1) and was performed using 1430 neutral SNPs with a minimum call rate of 0.8. Plots for the optimal number of clusters (Delta K) and genetic structure were made with *StructureSelector (Li and Liu 2018)* using *Clumpak* (Kopelman et al. 2015).

Figure S2: Genetic structure of 7 whitefish species collected from 7 Swiss lakes across 6 values (2-7) of Delta K. 1) *C.* sp. from Lake Joux (n = 10), 2) *C. confusus* from Lake Biel (n = 40), 3) *C. palaea* from Lake Geneva (n = 11), 4) *C. fatioi* from Lake Thun (n = 32), 5) *C. albellus* from Lake Brienz (n = 15), 6) *C. muelleri* from Lake Lucerne (n = 15), 7) *C. cf. suidteri* from Lake Hallwil (n = 92).

**Supplementary references**

Evanno G, Regnaut S, Goudet J. 2005. Detecting the number of clusters of individuals using the software structure: a simulation study. Molecular Ecology. 14(8):2611–2620. https://doi.org/10.1111/j.1365-294X.2005.02553.x

Falush D, Stephens M, Pritchard JK. 2003. Inference of Population Structure Using Multilocus Genotype Data: Linked Loci and Correlated Allele Frequencies. Genetics. 164(4):1567–1587. https://doi.org/10.1093/genetics/164.4.1567

Kopelman NM et al. 2015. Clumpak: a program for identifying clustering modes and packaging population structure inferences across K. Molecular Ecology Resources. 15(5):1179–1191. https://doi.org/10.1111/1755-0998.12387

Li Y-L, Liu J-X. 2018. StructureSelector: A web-based software to select and visualize the optimal number of clusters using multiple methods. Molecular Ecology Resources. 18(1):176–177. https://doi.org/10.1111/1755-0998.12719

Pritchard JK, Stephens M, Donnelly P. 2000. Inference of Population Structure Using Multilocus Genotype Data. Genetics. 155(2):945–959. https://doi.org/10.1093/genetics/155.2.945
